# Genome-wide interaction study reveals age-dependent determinants of responsiveness to inhaled corticosteroids in individuals with asthma

**DOI:** 10.1371/journal.pone.0229241

**Published:** 2020-03-02

**Authors:** Amber Dahlin, Joanne E. Sordillo, Michael McGeachie, Rachel S. Kelly, Kelan G. Tantisira, Sharon M. Lutz, Jessica Lasky-Su, Ann Chen Wu

**Affiliations:** 1 Channing Division of Network Medicine, Brigham and Women’s Hospital and Harvard Medical School, Boston, Massachusetts, United States of America; 2 Department of Population Medicine, PRecisiOn Medicine Translational Research (PROMoTeR) Center, Harvard Medical School and Harvard Pilgrim Health Care Institute, Boston, Massachusetts, United States of America; 3 Division of Pulmonary and Critical Care Medicine, Brigham and Women’s Hospital, Boston, Massachusetts, United States of America; Illumina Inc, UNITED STATES

## Abstract

While genome-wide association studies have identified genes involved in differential treatment responses to inhaled corticosteroids (ICS) in asthma, few studies have evaluated the potential effects of age in this context. A significant proportion of asthmatics experience exacerbations (hospitalizations and emergency department visits) during ICS treatment. We evaluated the interaction of genetic variation and age on ICS response (measured by the occurrence of exacerbations) through a genome-wide interaction study (GWIS) of 1,321 adult and child asthmatic patients of European ancestry. We identified 107 genome-wide suggestive (P<10^−05^) age-by-genotype interactions, two of which also met genome-wide significance (P<5x10^-08^) (rs34631960 [OR 2.3±1.6–3.3] in *thrombospondin type 1 domain-containing protein 4* (*THSD4*) and rs2328386 [OR 0.5±0.3–0.7] in *human immunodeficiency virus type I enhancer binding protein 2* (*HIVEP2*)) by joint analysis of GWIS results from discovery and replication populations. In addition to *THSD4* and *HIVEP2*, age-by-genotype interactions also prioritized genes previously identified as asthma candidate genes, including *DPP10*, *HDAC9*, *TBXAS1*, *FBXL7*, and *GSDMB/ORMDL3*, as pharmacogenomic loci as well. This study is the first to link these genes to a pharmacogenetic trait for asthma.

## Introduction

Asthma, the most common chronic illness in childhood, costs over $50 billion annually in the United States.[1–3] Inhaled corticosteroids (ICS) are the most effective asthma controller medications leading to significant symptom improvement for most patients; however, approximately a third of individuals respond minimally or not at all.[4] As the genetics of childhood-onset asthma appear to be distinct from adult-onset asthma[5], different genetic mechanisms may mediate childhood-onset versus adult-onset asthma. Furthermore, childhood-onset vs. adult-onset asthma tends to respond better to ICS treatment.[6] In addition, national guidelines for asthma suggest distinct management approaches for childhood-onset versus adult-onset asthma.[7]

Most studies in asthma pharmacogenetics combine data from adult and pediatric populations, despite the fact that, both clinically and phenotypically, manifestation of adult and pediatric asthma can vary widely in presentation.[8–10] Nevertheless, known associations between genes and asthma drug responses suggest that age-specific genetic mechanisms can regulate response to ICS. For example, *FCER2* was demonstrated to help predict response to ICS in multiple studies of children, yet there are no reports of the role of *FCER2* in ICS response in adults.[11–13] Because the distribution, number, and type of genetic polymorphisms capable of predicting asthma treatment responses may vary with changes in asthma phenotypes resulting from age, understanding how age impacts pharmacogenetic traits is important for improving treatment outcomes for patients.

The objective of this study was to identify single nucleotide polymorphisms (SNPs) that are associated with response to ICS by evaluating age-by-genotype interactions. We hypothesized that by accounting for age-by-genotype interactions, we would identify novel risk loci that predict age-specific responses to ICS in individuals with asthma.

## Subjects, materials and methods

### Study populations

Five independent cohorts inclusive of pediatric and adult asthma patients of European ancestry were evaluated (total sample size = 1,321). The pediatric asthma population included ICS treatment arms within the Childhood Asthma Management Program (CAMP),[14] adolescent participants from the Asthma Clinical Research Network (ACRN),[15] and two of the five trials in the Childhood Asthma Research and Education (CARE) cohort: the Pediatric Asthma Controller Trial (PACT)[16] and Characterizing Response to Leukotriene Receptor Antagonist and Inhaled Corticosteroid (CLIC)[17] trials. The adult asthma cohort comprised subjects from ACRN, and data from two biorepositories linked to de-identified electronic health records from the PharmacoGenomic discovery and replication in very large POPulations (PGPop) cohorts: the Marshfield Clinic Personalized Medicine Research Project (PMRP)[18] and Vanderbilt University Medical Center’s BioVu program (BioVu)[19]. A description of the samples from these populations used in this study is provided in S1 Table. Individuals who were present in more than one study population were removed prior to evaluation. All study procedures were approved by the respective Institutional Review Boards of each consortium and the Brigham and Women’s Hospital (the Partners Human Research Committee (PHRC)). Human Subjects approval was obtained from Partners Human Research Internal Review Board, Protocol #: 2002P000331. Written informed consent was obtained.

### Phenotyping and selection of cases and controls

The main outcome for this study was a dichotomized variable for ICS response, wherein poor response (cases) was defined by one or more asthma exacerbations while on ICS and good response (controls) was defined by absence of exacerbations while on ICS. An asthma exacerbation was defined as an emergency department (ED) visit or hospitalization due to asthma, or the need for oral corticosteroids (bursts), and was assessed during the respective study period for each cohort. From a total sample size of 1,321 subjects, we selected 407 cases and 376 controls (n = 783) from CARE, ACRN, and BioVU as a discovery population, and an additional 287 cases and 251 controls (n = 538) from CAMP and PMRP for replication. We also evaluated age as an interaction variable for ICS response in GWIS models. To account for outliers as a result of the extreme influences in age ranges and right skewing in the distribution of age, we transformed age (in years) using a quantile-normalized transformation. Demographic information for cases and controls in each population is summarized in Table 1.

**Table 1:** Demographics of study populations.

	Discovery (N = 783)			Replication (N = 538)	
	*CARE (N = 150)*	*ACRN (N = 220)*	*BioVu (N = 413)*	*CAMP (N = 176)*	*PMRP (N = 362)*
**Cases**					
Sample size (N)	113	24	270	58	229
Sex (% male)	58.4	29.2	27.4	69	29.7
Age*, yrs. (mean [range])	12.6 [9.2–17.2]	35.8 [14.0–55.8]	34.8 [20.0–68.5]	7.3 [2.3–15.8]	24.1 [5–46]
BMI (mean, [range])	20.9 [14.5–38]	25.2 [18.8–35.7]	31.2 [17.1–45.3]	18.5 [14.2–28.5]	26.9 [8.4–56.1]
**Controls**					
Sample size (N)	37	196	143	118	133
Sex (% male)	54.1	46.2	32.9	60.2	29.3
Age*, yrs. (mean [range])	13.4 [10.0–16.8]	33.2 [14.4–65.2]	31.4 [20.0–65.5]	11.3 [6–17.8]	25.6 [8–44]
BMI (mean, [range])	21.7 [16.3–41.1]	25.2 [17.0–41.6]	28.7 [16.5–43.3]	20.7 [13.6–46.2]	26.1 [15.4–49.5]

*Age refers to age at event, for cases in PMRP and BioVu, or age at the final study visit, for all controls, and for the cases in CARE, ACRN and CAMP.

### Genotyping, imputation, and quality control procedures

Genotyping of DNA samples from subjects enrolled in the five study populations was previously described.[14–19]. To account for differences in the genotyping arrays and platforms used for each individual study, genetic markers across all five populations were merged using PLINK v.1.94[20, 21] (https://www.cog-genomics.org/plink2), pre-phased using Shape-IT v2.,5[22] and imputed to the 1000 Genomes Project (phase 1 integrated release[23]) reference CEU panels with IMPUTE2[24] (http://mathgen.stats.ox.ac.uk/impute/impute_v2.html). Prior to imputation, genetic markers with a minor allele frequency (MAF) < 1% and a genotype call rate < 90% were excluded, and a threshold for imputed SNPs was applied to exclude SNPs with imputation quality scores < 0.9 and MAF < 1%. Standard QC procedures were applied to the merged, imputed dataset using PLINK v.1.94 to remove samples and markers with below-threshold genotype and sample call rates, low minor allele frequency (<1%), and HWE *P* <10^−05^. Principal components analysis (PCA) was performed using PLINKv.1.94 to exclude individuals with significant non-European ancestry. A final dataset of 8,589,102 typed and imputed markers in 1,321 samples passed all sample and genotype QC measures for analysis.

### Statistical analyses

Genome-wide interaction studies (GWIS) were performed in the discovery (CARE, ACRN and BioVU; n = 783) and replication (CARE and PMRP; n = 538) populations, using PLINK v.1.94. The primary analysis tested for an age-by-genotype interaction as the outcome, using logistic regression models adjusted for the main effects of age, genotype, and covariates (gender, BMI, study, and the first six principal components). To assess the significance of identified interactions, we applied statistical significance thresholds that are routinely used for genome-wide association studies. We specified a genome-wide significance threshold of 5x10^-08^, while we also applied a more liberal genome-wide suggestive threshold of 1x10^-05^ to include interactions with P-values that were slightly above genome-wide significance but that may represent genuine interactions. For the replication GWIS, interactions meeting a P-value significance threshold of 0.05 (nominal significance) were included in the joint analysis. Following the discovery GWIS, age-by-genotype interactions were filtered based on meeting both genome-wide suggestive significance (P<1x10^-05^) in the discovery population as well as a nominal significance threshold (P<0.05) in the replication population. For replication, the P values for any age-by-genotype interactions that met the significance thresholds for both the discovery and replication populations and which also had concordant directionality of effect estimates for the age-by-genotype interaction variable were then combined using a weighted Z-score method.[25] Quantile-quantile plots of SNP associations demonstrated no significant inflation as a result of population stratification (S1 Fig).

### Post-hoc power calculations

We conducted a post-hoc power analysis to detect age-by-genotype interactions with the binary outcome for the most significant signal, rs34631960. To run an empirical power analysis for a gene by environment interaction with a binary outcome and a normally distributed environmental exposure, we created an R package on github called powerGcE (https://github.com/SharonLutz/powerGcE). Using estimates from our study, we generated a binomial distribution for a SNP with a MAF of 0.49, the minor allele frequency of rs34631960 in our population. The transformed age variable was generated from a normal distribution with a mean of 0 and a variance of 1. Then, the binary outcome was generated using estimates from our study such that

$$\text{logit}\left[\text{P}\left(\text{Y}=1\right)\right]=-0.32+0.17*\text{SNP}+0.97*\text{E}+\beta_{\text{I}}*\text{E}*\text{SNP}$$

where β_I_ varies from -1 to -0.75 by 0.05 since β_I_ was estimated to be 0.8 in our study. We set the significance threshold at the genome-wide significance threshold of P<5x10^-08^. The empirical power was then calculated based on the proportion of simulations where the P-value for the interaction term in a logistic regression was less than the user specified alpha level of 5x10^-08^. As seen in S2 Fig, for 10,000 simulations, we had adequate power for our discovery population with 407 cases and 376 controls to detect an age by genotype interaction for rs34631960.

## Results

**Fig 1:**
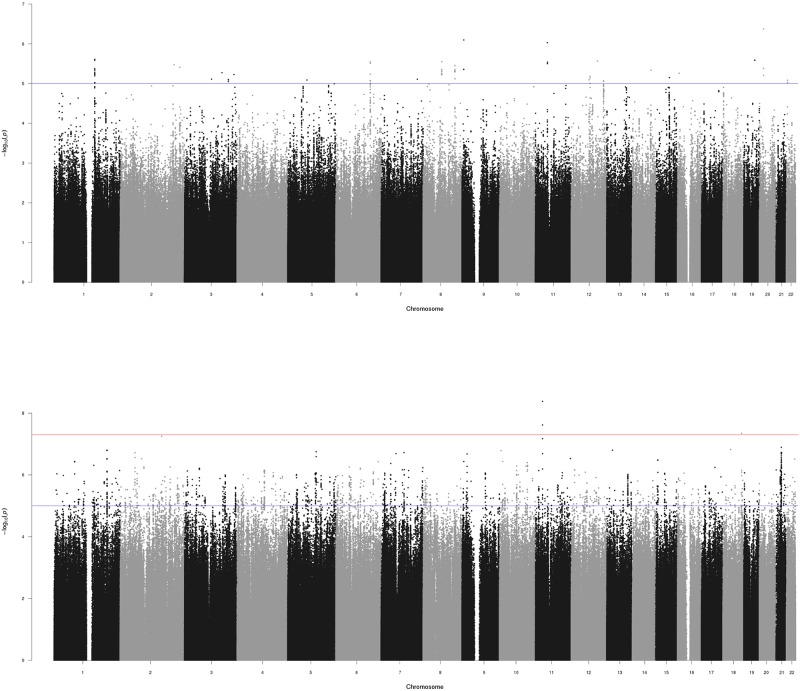
Results of age-by-genotype interaction study for ICS response in asthma. Manhattan plots show results for discovery (top panel) and replication (bottom panel) GWIS, by chromosome. Red line indicates threshold for genome-wide significance (P<5x10^-08^) while blue lines indicate suggestive threshold for genome-wide significance (P<10^−05^).

Our imputed datasets contained > 8 million SNPs in 1,321 pediatric and adult patients with asthma who were taking ICS. We evaluated age-by-genotype interactions with the outcome of ICS response in two independent (discovery and replication) GWIS. The discovery GWIS of 407 asthmatic cases with poor ICS response and 376 asthmatic controls with good ICS response (total n = 783; Table 1) identified 230 age-by-genotype interactions that were suggestive of genome-wide significance (P<1x10^-05^) (Table 2); however, none achieved genome-wide significance (P<5x10^-08^) (Fig 1). Among the suggestive associations, 107 SNPs were also at least nominally significant in the replication population. Of these, two SNPs (rs34631960 and rs2328386) achieved genome-wide significance by joint analysis (Table 2). Sixty-nine of the 107 replicated GWIS SNPs were localized to intergenic regions of chromosomes 2, 5, 11, and 12, while the remainder were distributed across eight unique genes in chromosomes 1 (*SPRR2G* and *SAMD13*), 6 (*HIVEP2*), 8 (*SAMD12*), 15 (*THSD4*), and 16 (*RBFOX1*). All but one of the genic SNPs were predicted to reside within the introns of their respective genes, while rs509194 was annotated to a 3’UTR in *SPRR2G*.

**Table 2:** Summary of top 20 replicated age-by-genotype interactions obtained through GWIS of ICS response.

					Discovery		Replication		
SNP	Chr.	Major Allele	Minor Allele	Gene	Odds Ratio (95% CI)	P Value	Odds Ratio (95% CI)	P Value	Joint P Value*
rs34631960	15	CTT	C	*THSD4*	2.33(1.61–3.38); 0.12 (0.02–0.85)∞	7.08X10^-06^	1.82(1.23–2.7); 7.35 (1.23–44.05)∞	2.97X10^-03^	3.64X10^-08^
rs2328386	6	C	T	*HIVEP2*	0.33(0.2–0.55); 0.19 (0.02–1.41)∞	1.86X10^-05^	0.51(0.34–0.77); 0.55 (0.07–4.28)∞	1.49X10^-03^	4.98X10^-08^
rs290119	5	G	A		2.38(1.6–3.53); 0.27 (0.03–2.51)∞	1.76X10^-05^	1.96(1.28–3); 16.6(0.64–433.2)∞	1.99X10^-03^	6.13X10^-08^
rs290122	5	C	T		2.38(1.6–3.53); 0.27 (0.03–2.51)∞	1.76X10^-05^	1.96(1.28–3); 16.6(0.64–433.2)∞	1.99X10^-03^	6.13X10^-08^
rs58836160	5	T	TA		2.38(1.6–3.53); 0.27 (0.03–2.51)∞	1.76X10^-05^	1.96(1.28–3); 16.6(0.64–433.2)∞	1.99X10^-03^	6.13X10^-08^
rs6892109	5	C	T		2.7(1.73–4.22); 0.01 (0–0.24)∞	1.34X10^-05^	2.09(1.26–3.46); 6.2(0.38–101.5)∞	4.39X10^-03^	9.90X10^-08^
chr12:121988899	12	C	T		2.38(1.62–3.48); 0.32 (0.05–2.2)∞	8.52X10^-06^	1.69(1.14–2.51); 2.6(0.01–474.3)∞	9.73X10^-03^	1.42X10^-07^
rs12658947	5	A	G		2.69(1.72–4.2); 0.02 (0–0.26)∞	1.51X10^-05^	2.01(1.21–3.34); 4.84(0.3–79.23)∞	6.71X10^-03^	1.69X10^-07^
rs12659412	5	T	C		2.69(1.72–4.2); 0.02 (0–0.26)∞	1.51X10^-05^	2.01(1.21–3.34); 4.84(0.3–79.23)∞	6.71X10^-03^	1.69X10^-07^
rs509061	1	T	C	*SPRR2G*	2.08(1.5–2.89); 1.43 (0.33–6.26)∞	1.28X10^-05^	1.72(1.15–2.59); 1.34(0.18–10.13)∞	8.50X10^-03^	1.82X10^-07^
rs2052548	5	A	C		2.73(1.74–4.27); 0.01 (0–0.26)∞	1.18X10^-05^	1.93(1.17–3.2); 3.64(0.25–52.65)∞	1.02X10^-02^	2.03X10^-07^
rs72755734	5	A	G		2.72(1.74–4.26); 0.01 (0–0.25)∞	1.22X10^-05^	1.93(1.17–3.2); 3.64(0.25–52.65)∞	1.02X10^-02^	2.09X10^-07^
rs1477347	5	G	A		2.71(1.73–4.24); 0.01 (0–0.25)∞	1.31X10^-05^	1.93(1.17–3.2); 3.68(0.25–53.25)∞	1.02X10^-02^	2.24X10^-07^
chr12:121973317	12	G	A		2.30(1.58–3.35); 0.35 (0.05–2.33)∞	1.45X10^-05^	1.67(1.13–2.49); 2.62(0.01–477.1)∞	1.06X10^-02^	2.57X10^-07^
rs72755727	5	A	T		2.67(1.71–4.17); 0.01 (0–0.25)∞	1.72X10^-05^	1.94(1.17–3.21); 7.09(0.54–93.35)∞	1.00X10^-02^	2.84X10^-07^
rs77668680	1	C	CAG	*SPRR2G*	2.23(1.58–3.14); 1.87 (0.4–8.72)∞	4.23X10^-06^	1.54(1.02–2.33); 1.14(0.15–8.79)∞	4.16X10^-02^	3.81X10^-07^
rs6500715	16	G	C	*RBFOX1*	0.47(0.34–0.65); 0.59 (0.13–2.63)∞	5.51X10^-06^	0.69(0.49–0.97); 1.38(0.16–11.97)∞	3.54X10^-02^	3.96X10^-07^
rs34338452	8	AT	A	*SAMD12*	2.24(1.57–3.19); 1.02 (0.17–5.99)∞	7.58X10^-06^	1.59(1.05–2.39); 1.04(0.13–8.19)∞	2.78X10^-02^	3.99X10^-07^
rs10094604	8	G	T	*SAMD12*	2.28(1.6–3.24); 1.02 (0.17–6.02)∞	4.73X10^-06^	1.53(1.02–2.29); 1.13(0.15–8.72)∞	4.07X10^-02^	4.09X10^-07^
rs524887	1	A	G	*SAMD13*	2.23(1.58–3.14); 1.88 (0.4–8.75)∞	4.57X10^-06^	1.53(1.01–2.31); 1.04(0.14–8)∞	2.70X10^-02^	3.99X10^-07^

Odds ratios and 95% confidence intervals are provided for age-by-genotype interactions.

^∞^Odds ratios with 95% confidence intervals for the main effect estimates. Minor allele frequencies for all variants are > 1%.

*P values were combined using a weighted Z-score method.

The top two SNPs, rs34631960 and rs2328386, were localized to *thrombospondin type 1 domain-containing protein 4 (THSD4)* and *human immunodeficiency virus type I enhancer binding protein 2* (*HIVEP2*), respectively (Table 2). rs34631960 was associated with an approximately 2-fold increase in risk of exacerbations on ICS with age, while rs2328386 was associated with a 0.3–0.5-fold decrease in risk of exacerbations on ICS with age (Table 2).

We also investigated age-by-genotype interactions for candidate genes within inflammatory pathways which were previously identified in studies of ICS response or asthma susceptibility. These genes included *dipeptidyl-peptidase 10* (*DPP10)*, *histone deacetylase 9* (*HDAC9)*, *thromboxane A synthase 1 (TBXAS1)*, *F-box and leucine rich repeat protein 7 (FBXL7)*, and *gasdermin B (GSDMB*/*ORMDL sphingolipid biosynthesis regulator 3 (ORMDL3)* locus. All genes had at least nominally significant SNP annotations in both the discovery and replication results; when ranking these candidate gene variants by the lowest P-values, *DPP10* was the top gene in the discovery set, while *HDAC9* was the top gene in the replication set (S2 Table). The top SNPs in both discovery and replication sets for *DPP10* were associated with lower risk of exacerbations on ICS with age, which was also observed for the top SNPs in *HDAC9* for both sets (S2 Table).

## Discussion

Our study demonstrates three key findings. First, the age-by-genotype interaction analysis identified two SNPs, rs34631960 in *THSD4* and rs2328386 in *HIVEP2*, that met genome-wide significance based on meta-analysis in 1,321 pediatric and adult patients with asthma who were taking ICS. To our knowledge, this is the first genome-wide study to link *THSD4* and *HIVEP2* to a pharmacogenomic outcome for asthma. Secondly, we identified additional novel age-by-genotype interactions related to ICS response. Lastly, our study underscores the importance of investigating age-by-genotype interactions in asthma.

The top-ranked age-by-genotype association (*THSD4* SNP rs34631960) could confer a protective effect on exacerbations risk in younger asthmatics taking ICS, or, conversely, may predict an increased risk of poor ICS response with increasing age. *THSD4* is potentially involved in regulating normal and abnormal lung function, angiogenesis, and airway remodeling in asthma and may also contribute to the progression to asthma, asthma severity, and exacerbations through these processes.[26–28] *THSD4* was also previously associated with COPD,[29] and also with measures of lung function in individuals with and without asthma, including FEV_1_/FVC ratio in large meta-analyses of individuals of European ancestry.[30–33] Consistent with these findings, our results demonstrate the association of *THSD4* with asthma exacerbations and further suggest a role for variation within *THSD4* as a potential mecahnism for increased risk of exacerbations with age, despite ICS use. There is little evidence to suggest that *THSD4* may be regulated by corticosteroid exposure. However, we observed that *THSD4* was differentially expressed in response to dexamethasone treatment of lymphoblastoid cell lines (LCLs) from individuals with asthma, although the [log_2_]_abs_ fold change in expression was below threshold (data not shown). Additional studies are warranted to discern the mechanistic role(s) of *THSD4* in ICS response.

rs2328386 confers a C>T change in the large, zinc-finger containing transcription factor, *HIVEP2*. Like rs34631960, rs2328386 was also annotated to an intronic region; therefore, the SNP may be unlikely to have direct functional effects. *HIVEP2*, also known as *Schnurri (Shn)-2*, contributes to asthma pathogenesis through its role as an NFkB binding protein, and regulates Th2 cell differentiation and responses[34]. Furthermore, *HIVEP2* is also a negative regulator of Th2-mediated allergic airway inflammation; Shn-2 deficient mice demonstrated increased eosinophilic inflammation, mucus hyperproduction and airway hyperresponsiveness.[34] *HIVEP2* is likely to be involved in other asthma phenotypes, as it mediates multiple aspects of growth and development for a variety of biological pathways and tissues, notably the nervous system,[35] immune response pathways,[36–38] and bone remodeling.[39] *HIVEP2* could potentially contribute to asthma severity and drug response through glucocorticoid receptor activity; *Hivep2* expression is inducible in dexamethasone-stimulated, mouse bone-marrow-derived macrophages, via glucocorticoid receptor-mediated effects on chromatin reorganization.[40] Furthermore, as it also binds to the enhancers of multiple genes in the MHC region of chromosome 6, *HIVEP2* regulates the activity of a variety of additional genes involved in immune responses, inflammation and glucocorticoid response.[41,42] Variation in *HIVEP2* has also been noted as a contributing factor for neurodevelopmental disorders, such as schizophrenia, and *HIVEP2* is thought to promote the activation of inflammatory pathways precipitating these diseases.[43] Our findings suggest that *HIVEP2* variation may also contribute to age-dependent corticosteroid response in asthma patients.

To date, multiple genes that influence allergic inflammatory pathways have been associated with asthma phenotypes including *DPP10*, which is implicated in asthma susceptibility,[44, 45] *HDAC9*, which is associated with asthma severity,[46] *TBXAS1* and *FBXL7*, which are associated with ICS response in children,[47] and the *GSDMB*/*ORMDL3* locus, which is associated with child-onset asthma and is also influential for ICS response.[48] Due to their importance in inflammatory pathways related to asthma phenotypes, we tested for association of these genes in the age-by-genotype interaction results using the top-ranked SNP within 10 kb of each candidate gene as a proxy, and identified *DPP10* and *HDAC9* as the most significantly associated genes for the discovery and replication datasets. The top SNPs in both genes were associated with lower risk of exacerbations while taking ICS, in both cohorts (S2 Table). While *DPP10* and *HDAC9* were implicated as risk loci for asthma susceptibility and/or severity and related phenotypes, this study is the first to indicate an association of these genes with ICS response. Furthermore, we confirmed previously reported associations of *TBXAS1*, *FBXL7*, and *GSDMB/ORMDL3* with ICS response, and also contribute several new age-by-genotype interactions to this list.

Strengths of this study comprise the inclusion of multiple clinical trials and ‘real-life’ asthma populations of diverse age ranges, spanning childhood, adolescence, and adulthood. To our knowledge, no previous pharmacogenetic studies have specifically examined the effect of age-by-genotype interactions on the outcome of differential ICS responsiveness, as measured by the occurrence of exacerbations concurrent with ICS use. However, we acknowledge that there are limitations with the selection of exacerbations as a pharmacogenomic phenotype for ICS response, because in addition to patient genetics, other important factors may also contribute to exacerbations on ICS, such as medication adherence, environmental conditions, and disease-related co-morbidities. The cohorts investigated in this study were derived from well-characterized asthma clinical trials and biorepositories, and we excluded patients with respiratory-related comorbidities that might influence exacerbations, such as COPD, bronchiectasis, and pulmonary hypertension. Combining heterogeneous data from different clinical study and patient populations is further hindered by the fact that additional relevant information including ICS dose, atopy status, etc., is not universally available across studies. While it was difficult to ascertain the extent of ICS adherence or dose (exposure) in these populations, as this information is not readily available across cohorts, to avoid including subjects with poor adherence, our study populations were restricted to patients with an EMR of concurrent ICS prescription throughout the two-year study window. We attempted to minimize bias introduced by combining data from multiple studies, through standardizing the definition of exacerbations across studies and by merging genotype data (which was originally generated using different array platforms). Further, while we sought to capture ages of the cases reported at the time of the exacerbations event, in the absence of this information, we analyzed age as recorded at the two-year study visit instead, which was appropriate given that exacerbations were evaluated cumulatively through this time point. Despite this, we acknowledge that differences across studies and populations could represent a limitation. In addition, our study was restricted to subjects of European ancestry due to the larger sample sizes available for investigation, which could limit generalizability of our findings to the asthma patient population. As a result, future age-by-genotype analyses in diverse populations are warranted. While the causes of exacerbations are multi-factorial, our study design is tailored to capture specific genetic associations with the ICS response outcome as modified by age, in patients who are taking ICS. However, as we evaluated only ICS treated asthma patients (vs. a non-ICS treated group), we acknowledge that it is difficult to discern if the interactions we identified reflect those that differ by age in both ICS treated and untreated populations. Further, our sample sizes may not be large enough to detect certain interactions of lower significance levels that could potentially contribute to the phenotype; however, the sample size of our population is relatively large for pharmacogenomics GWAS, particularly of asthma, and post-hoc power analyses showed that our study was adequately powered (S2 Fig). While we did not identify genome-wide significant associations in the discovery analysis, we were able to identify associations that reached genome-wide significance through joint analysis of both discovery and replication populations. As the top age-by-genotype interactions were annotated to introns of *THSD4* and *HIVEP2*, they are unlikely to confer direct functional effects with regard to the phenotype. Furthermore, neither SNP is an eQTL in the Genotype-Tissue Expression (GTEx) portal (https://gtexportal.org/home/) for any gene or tissue. Despite this, the present study, in addition to previous literature defining roles for both *THSD4* and *HIVEP2* in asthma, show that these may represent candidate genes for which additional molecular studies are warranted in order to clarify their roles in asthma and differential ICS response.

In summary, we have identified novel, age-dependent genetic polymorphisms associated with ICS response in adult and pediatric asthma patients. We report that *THSD4*, *HIVEP2*, *DPP10*, *HDAC9* and other genes within inflammatory response pathways and with definitive roles in asthma susceptibility, severity, and exacerbations, are also asthma pharmacogenes. These findings contribute to precision medicine-driven efforts to surmount treatment-resistant asthma in patients of all ages.

## Supporting information

S1 Fig Quantile-quantile plots of GWIS for (A) discovery and (B) replication. (DOCX)

S2 Fig (DOCX)

S1 Table Summary of clinical trials and patient populations. (DOCX)

S2 Table Enrichment of candidate genes in age-by-genotype interaction studies. (DOCX)
