# Supplementary material for: Genome-wide interaction study reveals age-dependent determinants of responsiveness to inhaled corticosteroids in individuals with asthma
Source: PLoS One. 2020 Mar 2;15(3):e0229241. doi: 10.1371/journal.pone.0229241 (PMC7051058; doi:10.1371/journal.pone.0229241)
Supplement: S1 Fig — (DOCX) [file pone.0229241.s001.docx]

**Supplemental Figure 1.** Quantile-quantile plots of GWIS for (A) discovery and (B) replication.

**Supplemental Figure 1.**

1. B.
