# Supplementary material for: Genome-wide interaction study reveals age-dependent determinants of responsiveness to inhaled corticosteroids in individuals with asthma
Source: PLoS One. 2020 Mar 2;15(3):e0229241. doi: 10.1371/journal.pone.0229241 (PMC7051058; doi:10.1371/journal.pone.0229241)
Supplement: S2 Fig — (DOCX) [file pone.0229241.s002.docx]

**Supplemental Figure 2.**

We ran a power analysis to detect the SNP by age interaction on the binary outcome for the most significant signal rs34631960 on chromosome 15. To run an empirical power analysis for a gene by environment interaction with a binary outcome and a normally distributed environmental exposure, we created an R package on github called powerGcE (https://github.com/SharonLutz/powerGcE). Using estimates based on our study, we generated a SNP from a binomial distribution with a MAF of 0.49, the minor allele frequency of the SNP in our population. The transformed age variable was generated from a normal distribution with a mean of 0 and a variance of 1. Then, the binary outcome was generated using estimates from our study such that

logit[P(Y=1)] = -0.32 + 0.17*SNP + 0.97*E + β_I_ *E*SNP

where β_I_ varies from -1 to -0.75 by 0.05 since β_I_  was estimated to be 0.8 in our study. Then, the empirical power was calculated based on the proportion of simulations where the p-value for the interaction term in a logistic regression was less than the user specified alpha level of 5E-8. As seen in the plot below for 10,000 simulations, we had adequate power for our discovery population with 407 cases and 376 controls to detect an age by SNP interaction for rs34631960.
