# Supplementary material for: Genome-wide interaction study reveals age-dependent determinants of responsiveness to inhaled corticosteroids in individuals with asthma
Source: PLoS One. 2020 Mar 2;15(3):e0229241. doi: 10.1371/journal.pone.0229241 (PMC7051058; doi:10.1371/journal.pone.0229241)
Supplement: S1 Table — (DOCX) [file pone.0229241.s003.docx]

**Supplemental Table 1. Summary of Clinical Trials and Patient Populations.**

| **Trial** | **Description of Subjects and Samples (N selected for GWIS profiling)^*^** | **Mean Age,yrs. (% children (<18 yrs.))** | **% Exacerbations^**^** | **Array Platform** |
| --- | --- | --- | --- | --- |
| Childhood Asthma Management Program (CAMP) | Subjects randomly assigned to budesonide treatment arm (N=176) | 10.0 (100%) | 75% | Illumina 550kv3 or 610 beadchip |
| Childhood Asthma Research ad Education (CARE) | Subjects from two trials with prospective ICS treatment response outcome: PACT and CLIC (N=150) | 12.8 (100%) | 33% | Affymetrix 6.0 |
| The Asthma Clinical Research Network (ACRN) | Subjects from corticosteroid treatment arms (N=220) | 33.5 (3.6%) | 10.9% | Affymetrix 6.0 |
| BioVU | DNA biorepository linking >130K DNA samples with de-identified EHR (N=413) | 33.56 (24.0%) | 65.4% | Illumina OmniExpress Exome chip |
| Marshfield Clinic Personalized Medicine Research Project (PMRP) | DNA biorepository linking ~20K DNA samples with de-identified EHR (N=362) | 24.6 (0%) | 63% | Illumina OmniExpress Exome chip |
| ^*^All DNA samples and subjects were of European ancestry | | | | |
| ^**^ % exacerbations occurring within the sample size in second column | | | | |
