# Supplementary material for: Genome-wide interaction study reveals age-dependent determinants of responsiveness to inhaled corticosteroids in individuals with asthma
Source: PLoS One. 2020 Mar 2;15(3):e0229241. doi: 10.1371/journal.pone.0229241 (PMC7051058; doi:10.1371/journal.pone.0229241)
Supplement: S2 Table — (DOCX) [file pone.0229241.s004.docx]

**Supplemental Table 2.** Enrichment of Candidate Genes in Age-by-Genotype Interaction Studies.

| **Candidate Gene** | **Discovery** | | | | **Replication** | | | |
| --- | --- | --- | --- | --- | --- | --- | --- | --- |
|  | **Best SNP** | **Minor Allele** | **OR (95% CI)** | **Best SNP P Value** | **Best SNP** | **Minor Allele** | **OR (95% CI)** | **Best SNP P Value** |
| *DPP10* | rs11123284 | A | 0.46 (0.31-0.69) | 2.00E-04 | rs3029135 | A | 0.31 (0.18-0.56) | 8.06E-05 |
| *TBXAS1* | rs11305533 | C | 0.58 (0.41-0.82) | 1.92E-03 | rs62490128 | T | 0.43 (0.26-0.71) | 9.59E-04 |
| *FBXL7* | rs13161679 | T | 3.60 (1.07-12.17) | 3.93E-02 | rs28406060 | G | 0.17 (0.08-0.39) | 2.19E-05 |
| *ORMDL3/GSDMB* | rs870830 | A | 0.63 (0.43-0.93) | 1.84E-02 | rs8065520 | C | 0.35 (0.11-1.19) | 9.26E-02 |
| *HDAC9* | rs79524815 | G | 0.27 (0.11-0.66) | 4.24E-03 | rs73075260 | T | 0.30 (0.17-0.51) | 1.05E-05 |
